# Combined Non-Thermal Microbial Inactivation Techniques to Enhance the Effectiveness of Starter Cultures for Kimchi Fermentation

**DOI:** 10.4014/jmb.2310.10010

**Published:** 2023-11-20

**Authors:** Su-Ji Kim, Sanghyun Ha, Yun-Mi Dang, Ji Yoon Chang, So Yeong Mun, Ji-Hyoung Ha

**Affiliations:** 1 Hygienic Safety · Materials Research Group, World Institute of Kimchi, Gwangju 61755, Republic of Korea; 2 Department of Food Science and Technology, Chung-Ang University, Anseong 17546, Republic of Korea; 3 Fermentation Regulation Technology Research Group, World Institute of Kimchi, Gwangju 61755, Republic of Korea

**Keywords:** Kimchi, disinfection, fermentation, lactic acid bacteria, starter cultures

## Abstract

For quality standardization, the application of functional lactic acid bacteria (LAB) as starter cultures for food fermentation is a well-known method in the fermented food industry. This study assessed the effect of adding a non-thermally microbial inactivated starter culture to kimchi, a traditional Korean food, in standardizing its quality. In this study, pretreatment based on sterilization processes, namely, slightly acidic electrolyzed water (SAEW) disinfection and ultraviolet C light-emitting diode (UVC-LED) of raw and subsidiary kimchi materials were used to reduce the initial microorganisms in them, thereby increasing the efficiency and value of the kimchi LAB starter during fermentation. Pretreatment sterilization effectively suppressed microorganisms that threatened the sanitary value and quality of kimchi. In addition, pretreatment based on sterilization effectively reduced the number of initial microbial colonies in kimchi, creating an environment in which kimchi LAB starters could settle or dominate, compared to non-sterilized kimchi. These differences in the initial microbial composition following the sterilization process and the addition of kimchi LAB starters led to differences in the metabolites that positively affect the taste and flavor of kimchi. The combined processing technology used in our study, that is, pre-sterilization and LAB addition, may be a powerful approach for kimchi quality standardization.

## Introduction

Kimchi was listed in the Codex Alimentarius (CODEX STAN 223-2001) in 2001 as a plant-based probiotic food that promotes health benefits like that of dairy probiotic foods. Commercial kimchi products are fermented by lactic acid bacteria (LAB) at a temperature of 0-6°C; LAB affects not only the functionality and preservation of kimchi but also the taste and flavors [1]. The generation of organic compounds due to the acidic properties of LAB and the resulting continuous decrease in pH enables the maintenance of kimchi freshness during storage and distribution. Various compounds produced by LAB, such as ethanol, carbon dioxide, mannitol, γ-aminobutyric acid, ornithine, bacteriocins, and oligosaccharides, contribute to the functionality, food preferences, and fermentation properties of kimchi [2, 3]. Various metabolites affect kimchi quality during fermentation; therefore, recently, there has been a demand for a food engineering approach for a quality standardization strategy for kimchi, facilitating its acceptance as a fermented food worldwide. The changing pattern of the microbial community in kimchi and other naturally fermented vegetables has been reported to be fairly predictable and reproducible [4]. However, quality standardization is required to more accurately predict various microbial communities in commercially available kimchi. For quality standardization, the application of LAB as functional starter cultures for food fermentation is a well-known method in the fermented food industry. Thus, depending on the functional starter culture used, various studies have investigated the influence of kimchi LAB starter inoculation on kimchi quality [5-7]. For example, *Leuconostoc citreum* produces substances that enhance bacteriocin activity [7], *Leuconostoc mesenteroides* increases mannitol levels [8, 9], and *Latilactobacillus sakei* [10] and *Pediococcus pentosaceus* produce lactic acid, contributing to extending the shelf life of kimchi [6]. Although several previous studies have verified that the addition of starter cultures contributes to food fermentation properties, none have investigated methods to improve the efficiency of starter culture addition.

Although the addition of specific single- or mixed-starter strains can contribute to fermentation, the dominance of the starters can change depending on the abundance of the environmental microbiota [11]. There are two important obstacles to the application of kimchi LAB starter cultures for the standardization of kimchi quality: (a) the dominance rate of the added kimchi LAB starter cultures cannot be guaranteed with the addition of the specific LAB as kimchi starter cultures because fermentation is markedly influenced by the natural microbiota population and (b) the lack of a strain-specific quantifiable detection technique prevents the qualitative and quantitative monitoring of kimchi LAB starter strains in kimchi products during fermentation.

One approach to ensure the dominance rate of kimchi LAB starter cultures is the use of non-thermal techniques to inactivate the natural microbiota of kimchi. Among other methods, sterilization treatment technology can be used to reduce the natural microbiota of kimchi. This approach reduces the kimchi microbiota population, thereby allowing kimchi LAB starters to gain advantages. Kimchi, composed of various microbiota, is a representative minimally processed and non-thermally processed vegetable fermented food. Therefore, to control the kimchi microbiota, technologies based on non-thermal sterilization are necessary. Recently, chemical disinfectant including electrolyzed water, sodium hypochlorite, ozonated water, and plasma-activated water has been used as inactivation agents for various vegetables [12, 13]. In addition, ultraviolet C-light emitting diode (UVC-LED) inactivation techniques based on nonthermal treatment for the reduction of harmful microorganisms have been recognized as acceptable disinfection processes for application in the food industry [14]. Song *et al*. [15] reported that UVC-LED combined with an impeller system enabled the inactivation of kimchi seasoning contaminated with spoilage yeast. Furthermore, UVC-LED irradiation could be a promising new practical alternative to limited antimicrobial activities for reducing the kimchi microbiota population and promoting the dominance of kimchi LAB starters.

The development of a strain-specific identification assay for tracking kimchi LAB starter strains is important for the qualitative and quantitative monitoring of specific microorganisms (kimchi LAB starter) in fermented kimchi. Another method for the rapid detection of specific microorganisms to assess the dominance rate of kimchi LAB starter strains is the polymerase chain reaction (PCR). Lee *et al*. [8, 9] developed a DNAzyme-based colorimetric assay to quantify the dominance rate of *Leu. mesenteroides* WiKim32 as a LAB starter in fermented kimchi. According to Lee *et al*. [8, 9], the high quantitative capacity and detection sensitivity of fermented kimchi do not require DNA extraction and isolation. Therefore, the developed technique is an effective, sensitive, and rapid detection assay with potential industrial applications.

This study aimed to evaluate the efficacy of non-thermal techniques for microbial inactivation in reducing the natural microbiota population of fresh raw materials to improve the efficiency of kimchi LAB starters. To assess this, quality monitoring was performed, including determining the dominance rate of kimchi LAB starters in the fermented kimchi sample during the fermentation period.

## Materials and Methods

### Kimchi Sample Preparation

Kimchi ingredients, including kimchi cabbage, radish, garlic, and other seasonings, were purchased from a local Kimchi-Town store in Gwangju, Korea (35° 03´ 13˝ N). To prepare the brined cabbage and kimchi samples, fresh kimchi cabbage was washed and salted using sea salt. Brining condition including temperature, concentration, and time were 19 C, 10%, and 15 h, respectively [16]. Immediately thereafter, kimchi seasoning was mixed. Kimchi groups were categorized according to the pre-treatment for microbial inactivation and/or addition of *Leu. mesenteroides* WiKim32 as a kimchi LAB starter culture. The four kimchi groups were labeled as follows: i) commercial kimchi (CK), ii) commercial kimchi supplemented with kimchi LAB starter without microbial inactivation (CK-S), iii) microbial inactivated kimchi without kimchi LAB starter (DK), and iv) microbial inactivated kimchi supplemented with kimchi LAB starter (DK-S). To prepare starter cultures, *Leu. mesenteroides* WiKim32 (approximately 6.00 log_10_ CFU/g) was added to kimchi seasoning, and the mixture of seasoning and starter cultures was evenly mixed with brined cabbage. Low-density polyethylene (LDPE) bags were used for fermentation storage. Fermentation temperature was maintained at 4.0 ± 1.0°C for 70 days.

### Non-Thermal Inactivation

For slightly acidic electrolyzed water (SAEW) treatment, SAEW a pH of 5.25 ± 0.25, an available chlorine concentration (ACC) of 38 ± 2 mg/ml, and an oxidation-reduction potential (ORP) of 998 ± 35 mV was produced using an electrolysis apparatus (Purester MP_600T; Morinaga Engineering Co., Japan) at 4.7 A and 12.1 V. A dual-scale pH/mV meter (Accumet AB15; Fisher Scientific, USA) equipped with pH and ORP electrodes was utilized to determine the pH and ORP of the test solutions. ACC was determined using a standard iodometric titration procedure [17]. SAEW treatment of salted kimchi cabbage was carried out by soaking and rinsing twice for 10 min.

To inactivate the environmental microbiota, a non-thermal treatment was performed by UVC-LED irradiation using a module with a UVC-LED printed circuit board (620 × 430 × 76 mm, PCB, LANICS Co., Ltd. Republic of Korea) with specific peak wavelengths, including UVC (a radiometer with a maximum sensitivity of 267 nm). A bench-top setup was constructed to reduce the microbiota population during kimchi seasoning. For UVC-LED irradiation, a UVC-LED printed circuit board placed in 72 arrays was fixed 30 mm above the surface of the kimchi seasoning. The average intensity of the collimated 270 ± 3 nm UVC-LED was calculated over the illuminated area required to decontaminate the kimchi seasoning. For irradiation, 200 g of kimchi seasoning was transferred to a square storage container (500 × 400 × 30 mm; Thermo Fisher Waltham, USA). A double 3-blade impeller was then placed in the center of the storage container and stirred consistently at 250 rpm for uniform sterilization at 100 μW/cm^2^. The UVC dose (mJ/cm^2^) was defined as the product of the incident intensity (μW/cm^2^) and exposure time (s). The irradiance of the UVC-LED module was determined using a photo radiometer (HD-2102.2, Italy). Non-thermal treatment was performed at 20 ± 1°C.

### Analysis of Viable Cell Counts in Raw Ingredients and Kimchi Samples

Sterile saline (0.85%, 225 ml) per 25 g of sample was mixed in a sterile stomacher bag (3M, USA) and homogenized for 3 min using a stomacher blender (BagMixer 400; Interscience, France). Each sample mixture was then diluted 10-fold with sterile 0.85% saline. Total aerobic bacteria, coliforms, and yeasts and molds were enumerated according to the Food Code (Ministry of Food and Drug Safety, Republic of Korea). For total aerobic bacteria count, kimchi samples were plated onto plate count agar (BD/Difco Co.) and incubated at 37°C for 48 h. For enumeration of the yeast and mold, kimchi samples were plated onto potato dextrose agar (PDA, BD/Difco Co.) with 10% tartaric acid. PDA plates were incubated at 24 ± 1°C for 70 h. For enumeration of coliform, kimchi samples were plated onto Petrifilm Coliform Count Plates (3M Co.) and incubated at 37°C for 24 h. For enumeration of LAB, diluted samples were plated onto modified de Man, Rogosa, and Sharpe agar (BD/Difco Co.) with 0.01% cycloheximide and 0.2% bromocresol purple indicator and incubated at 37°C for 48 h.

### Analysis of Physicochemical Value

The homogenized kimchi sample was filtered to investigate titratable acidity (TA) and pH values. pH was determined using a pH meter (Model Titro Line 5000; SI Analytics, Germany). The kimchi samples were titrated to a pH of 8.3 by the addition of 0.1 N sodium hydroxide (NaOH; Daihan Scientific, Republic of Korea) solution. The TA was calculated as the percentage of lactic acid using the following formula (Eq. (1)).

$$\text{Titratable acidity }(\%)=\frac{(TV\times T\times DF)\times 0.009}{S}\times 100$$ (1)

where *TV* is the titration volume (ml) of NaOH (0.1 N), *T* is the titer of 0.1 N NaOH, *DF* is the dilution factor of the sample, *S* is the sample weight (g), and 0.009 is the amount of lactic acid in 1 ml of NaOH (0.1 N).

The kimchi samples were ground and filtered three times through sterilized gauze filtered with a nylon membrane filter (0.45 μm) and used as a sample solution for free sugar analysis. A calibration curve was constructed by deriving standard peaks by injecting 6 μl each of the test and standard solutions into an HPLC column (1260-Infinity, Agilent Technologies, USA) equipped with a refractive index detector and calculating free sugar content in the test solution. The column was a carbohydrate column (Asahipak-NH2P-50-4E, Shodex, Japan) and the oven temperature was 30°C. The mobile phase consisted of 75% acetonitrile in deionized water at a flow rate of 1 ml/min. Free sugar content was estimated using standard curves for fructose, glucose, sucrose, maltose, and mannitol.

### Analysis of Volatile Compounds (VCs)

For VC extraction, the kimchi samples (approximately 1 g) were homogenized, mixed with distilled water (approximately 1 ml), placed in a headspace vial (20 ml, Gerstel, Germany), and incubated at 70°C for 20 min under agitation at 300 rpm. Subsequently, the equilibrated headspace VCs were adsorbed for 40 min using a solid-phase microextraction (SPME) fiber (57329-U, Supelco, USA) coated with divinylbenzene/carboxene/polydimethylsiloxane. The captured SPME fiber was injected into a gas chromatography (GC) injector using a multipurpose autosampler (MPS2; Gerstel, Germany) for 3 min at 250°C. Before extraction, SPME fiber was activated at 250°C (60 min) to eliminate impurities.

VCs were identified using a GC (Agilent 7890-A; Agilent Technologies, USA) attached with a mass spectrometer (5977-B; Agilent Technologies, USA). The VCs were separated using a DB WAX capillary column (0.24 mm × 59 m × 0.24 μm; Agilent) with helium (≥99.999%) gas at 1 ml/min flow rate and in splitless injection mode. The oven temperature program was initially at 40°C for 180 s, raised to 150°C at 2°C/60 s, held for 9 min, raised to 210°C at 4°C/60 s, and held for 10 min. The detector and ion source temperatures were 250°C and 230°C, respectively. The electron ionization method was set to 72 eV and the mass spectrometry scan ranged from 40-500 m/z. The total ion chromatogram was analyzed using Mass-Hunter qualitative analysis software (B07.00; Agilent) and a library (WILEY-10N) according to the mass spectra.

### *L. mesenteroides* WiKim32 Detection in Kimchi

Microorganisms in kimchi filtrates were identified by a standard plate count procedure [18]. The kimchi filtrates were serially diluted with a 0.85% (w/v) solution of physiological saline and spread on plate count agar (Difco, USA), deMan, Rogosa, and Sharpe (MRS) agar (Difco) plates containing 2% (w/v) CaCO_3_ for total viable bacteria, and LAB enumeration, respectively. The plates were incubated at 30°C for 48 h. The numbers of microorganisms detected were expressed as colony forming units (CFU)/ml. For determining the proportion of WiKim32 in starter kimchi, the colonies formed on the agar plates were identified by WiKim32-specific DNAzyme-based LAMP assays [9]. Genomic DNA was extracted from each colony using a genomic DNA prep kit for bacteria (Qiagen, Germany) [8, 9] and the Lamp assay was performed using the Loopamp kit (Eiken Chemical Company, Japan) with DNAzyme (2.5 pmol final concentration). The reaction mixture was prepared as described previously [9], and incubated at 64°C for 50 min and then at 95°C for 2 min for enzyme inactivation. For colorimetric analysis, 10 μl of LAMP product was visualized by adding 1.5 μl of 100 μM hemin and 100 μl of working buffer solution (citrate buffer (pH 4.0) with 20 mg/ml ABTS and 30% H_2_O_2_ (1571:143:1, v/v/v)). After incubation at room temperature for 20 min, the color shift was observed visually. The samples that remained colorless were confirmed as WiKim32.

### Microbial Community Analysis

The composition of the gut microbiota was determined by CJ Bioscience, Inc. (Korea). PCR amplification was performed using fusion primers targeting the V3 to V4 regions of the 16S rRNA gene in the extracted DNA. Bacterial amplification was performed using the fusion primers 341F (5'-TGATACGGCGACCACCGAGAT CTACACXXXXXXXXTCGTCGGCAG-CGTCAGATGTGTATAAGA GACAGCCTACGGGNGGCWGCAG-3') and 805R (5'-CAAGCAGAAGACGGCA-TACGAGAT-XGTCTCGTGGGCTCGGAGATGTGTATAAGAGAC AGACTACH-VGGGTATCTAATCC-3'). PCR was performed using Master Mix and PTC-200 Peltier Thermal Cycler (Applied Biosystems, USA) using the following program: initial denaturation at 94°C for 5 min, followed by 30 cycles of denaturation at 94°C for 30 s, annealing at 55°C for 45 s, and extension at 72°C for 90 s. Each PCR product was purified using a QIAquick PCR Purification Kit (Qiagen), and the refined PCR products were quantified using the Quant-iTTM PicoGreen dsDNA Assay Kit (Invitrogen, Austria). Equal amounts of each DNA sample were mixed, and electrophoresis was performed using the pooled DNA sample. After emulsion-based clonal amplification of the DNA library samples, sequencing was performed using the emPCR Amplification 7020 Thermal cycler (Applied Biosystems) using the following program: initial denaturation at 94°C for 4 min, followed by 50 cycles of denaturation at 94°C for 270 s, annealing at 58°C for 45 s, and extension at 68°C for 30 s. Data analysis was performed using the CLcommunity ver.3.46 software (CJ Bioscience Inc., Republic of Korea) and the EZBioCloud database.

### Statistical Analysis

All experiments were repeated three times (technical repeats). One-way analysis of variance (ANOVA) and Duncan's multiple range tests were conducted using the Statistical Package for the Social Science v.19 (SPSS Inc., USA). All data are expressed as the mean ± standard deviation. Differences at *p* < 0.05 were considered significant.

## Results and Discussion

### Non-Thermal Treatment for Microbial Inactivation of Raw Ingredients

The reduction in the microbial populations in the SAEW- and UVC-LED-treated raw ingredients of kimchi products is shown in Tables 1 and 2, respectively. The initial total aerobic populations in four kinds of fresh raw ingredients, namely, kimchi cabbage, radish, garlic, and ginger were 5.52 ± 0.52, 5.03 ± 0.23, 6.65 ± 0.11, and 7.66 ± 0.17 log_10_ CFU/g, respectively. The initial total aerobic bacteria (TAB) in brined cabbage, chili powder, and kimchi seasoning, was 4.01 ± 0.34, 5.85 ± 0.14, and 6.99 ± 0.18 log_10_ CFU/g, respectively. SAEW treatment for 5 min resulted in a reduction in TAB populations in kimchi cabbage and radish to3.07 and 2.79 log_10_ CFU/g, respectively. UVC-LED irradiation reduced TAB in chili powder and kimchi seasoning to 0.37 and 1.80 log_10_ CFU/g, respectively. The inactivation of yeasts/molds and total coliforms showed a reduction pattern similar to that of TAB, with high reduction values in kimchi cabbage, radish, and kimchi seasoning. SAEW is advantageous in that it offers a strong bactericidal effect even at a low available chlorine concentration, minimizing the risk of damage to fresh produce [19]. Moreover, previous studies have shown the applicability of SAEW for the effective inactivation of pathogens on the surfaces of fresh produce [20-22]. However, despite the relatively low chlorine concentration, SAEW still contains chlorine, which leads to the formation of chlorinated organic compounds that are potentially harmful to human health. As a precautionary measure, an additional washing step with water is necessary. SAEW treatment is gaining attention in the food industry for its antimicrobial effects. The bactericidal activity of SAEW had been shown to exhibit strongly inactivate pathogenic bacteria including *Staphylococcus aureus*, *Escherichia coli* O157:H7, *Yersinia enterocolitica*, *Listeria monocytogenes*, and *Salmonella enteritidis* on various produce [21]. Recently, UVC-LED irradiation for the reduction of harmful microbial populations has received considerable attention as an innovative technique in the food industry [14]. Song *et al*. [15] demonstrated UVC-LED-mediated inactivation of spoilage yeasts without causing significant changes in the physicochemical properties of kimchi seasoning. Our results of reduction effects on microbial populations in different raw ingredients are in line with those of a previous study [15]. Microbial activity in disinfected raw ingredients directly affects the microbial community of the final fermented product due to changes in the initial number of bacteria [23]. Considering that kimchi contains various raw ingredients, the natural translocation of diverse indigenous microbiomes can establish unique cooperative or competitive relationships between microbes during early fermentation and a specific dominant microbiota during late fermentation [24]. In conclusion, even if the raw ingredients were not completely sterilized after the SAEW treatment or UVC-LED irradiation, samples of each raw ingredient for kimchi for the kimchi LAB starter application test contained a remarkably reduced number of natural microbiota.

### Changes in Physicochemical Properties of Kimchi during Fermentation

**Fig. 2:**
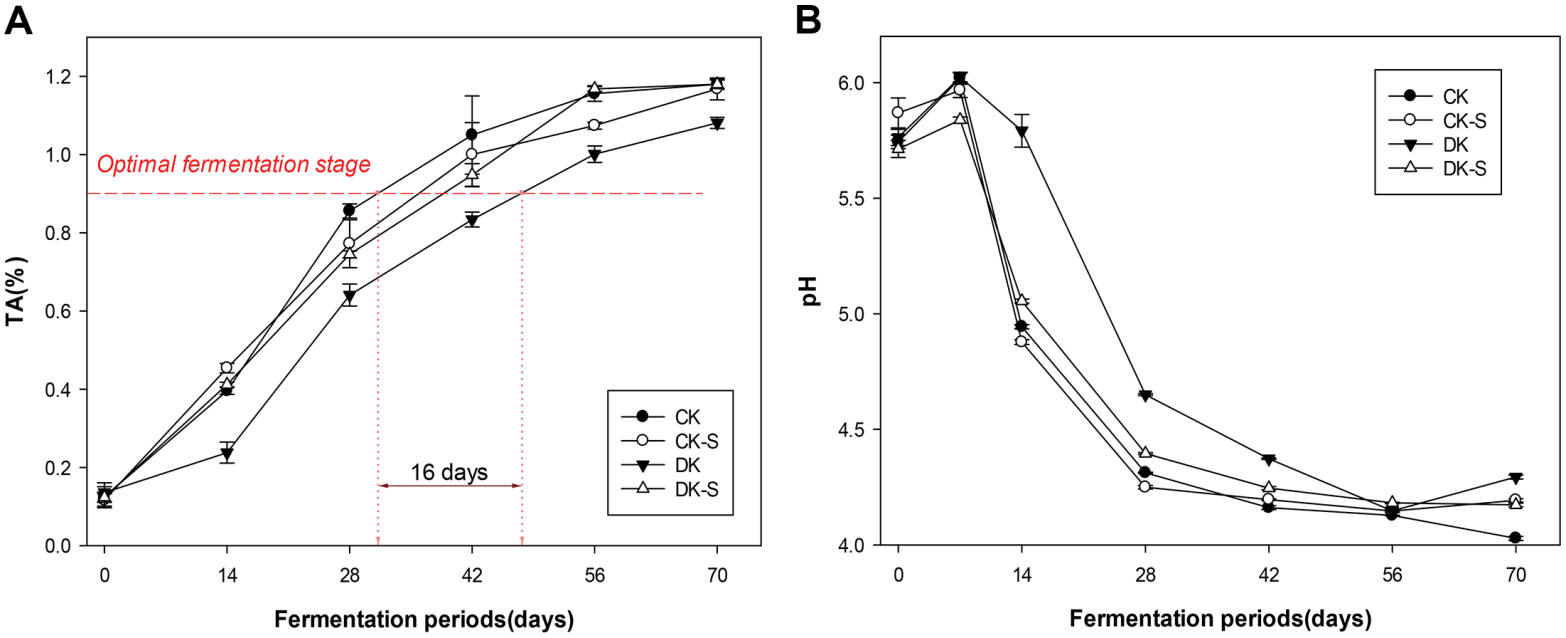
Changes in (A) pH values and (B) titratable acidity (TA) of the four kimchi groups stored at 4°C for 70 days: i) commercial kimchi (CK), ii) commercial kimchi supplemented with kimchi LAB starter without non-thermal treatment (CK-S), iii) microbial inactivated kimchi without kimchi LAB starter (DK), and iv) microbial inactivated kimchi supplemented with kimchi LAB starter (DK-S).

The changes in the TA and the pH values of the four groups of kimchi samples (CK, CK-S, DK, and DK-S) for 70 days during kimchi fermentation at 4°C were measured and are shown in Fig. 2. The average TA and pH values of the four groups ranged from 0.12% to 0.14% and from 5.71 to 5.87, respectively, and were not considerably different among the groups at the beginning of fermentation. As fermentation progressed, the TA increased in the CK, CK-S, and DK-S groups, ranging from 0.40% to 4.45% at 14 days, whereas that in the DK group was 0.24%(Fig. 2A). The changes in the pH values of the four groups are presented in Fig. 2B. Overall, the DK group showed a significant difference during the fermentation period, and the TA increase rate was lower than the other three groups. All groups showed a decrease in pH during fermentation, regardless of *Leu. mesenteroides* WiKim32 inoculation or non-thermal treatments. Interestingly, the initial pH values in CK, CK-S, and DK-S groups (5.71 to 5.87) decreased substantially to 4.94, 4.88, and 5.06, respectively, on day 14 of fermentation, whereas that in the DK group was 5.79 on day 14 and showed no significant difference from the initial pH (Fig. 2B). Therefore, the commercial kimchi group (CK), that in which commercial kimchi supplemented with *Leu. mesenteroides* WiKim32 without non-thermal treatment (CK-S), and that in which *Leu. mesenteroides* WiKim32 was added after non-thermal treatment (DK-S) entered the normal fermentation stage, whereas the group which was only microbial inactivated (DK) and not supplemented with *Leu. mesenteroides* WiKim32 showed a reduction in fermentation microorganisms surviving in the raw ingredients and exhibited the lag phase in fermentation. In the comparison between groups, there was a significant difference in the pH decrease rate of the DK group, which was slower than that of the CK, CK-S, and DK-S groups during the fermentation period of 14 to 42 days. The change in the lag time of TA at the beginning of fermentation had a significant effect on the quality retention period as fermentation progressed. Alterations in TA are key parameters for sensory quality that influence kimchi shelf-life decisions during fermentation [25]. Previous studies have reported a TA in the ranges of 0.80 and 0.90%, as the optimal fermentation stage which has a high correlation with the most tempting taste, flavor, and texture of fermented kimchi [26-28]. Cho *et al*. [26] demonstrated that fermented kimchi generates byproducts such as ethanol, lactic acid, acetic acid, carbon dioxide, and VCs during fermentation by numerous LAB. After the optimal fermentation stage (between 0.80 and 0.90%) of fermented kimchi, the sensory quality of kimchi, based on purchase preference from consumers, declines progressively because the values of fermentation by-products within the kimchi change and increases considerably, showing a positive correlation with TA during fermentation. Therefore, the DK group took longer to reach the TA of 0.8–0.9%, which is the optimal fermentation stage. In particular, a difference of approximately 14 days was observed between the CK and DK groups in reaching the TA of 0.8–0.9%. Therefore, the non-thermal treatment using UVC-LED or SAEW had a significant effect on extending the quality retention period of kimchi.

### Changes in Microbiological Composition in Kimchi during Fermentation

**Fig. 3:**
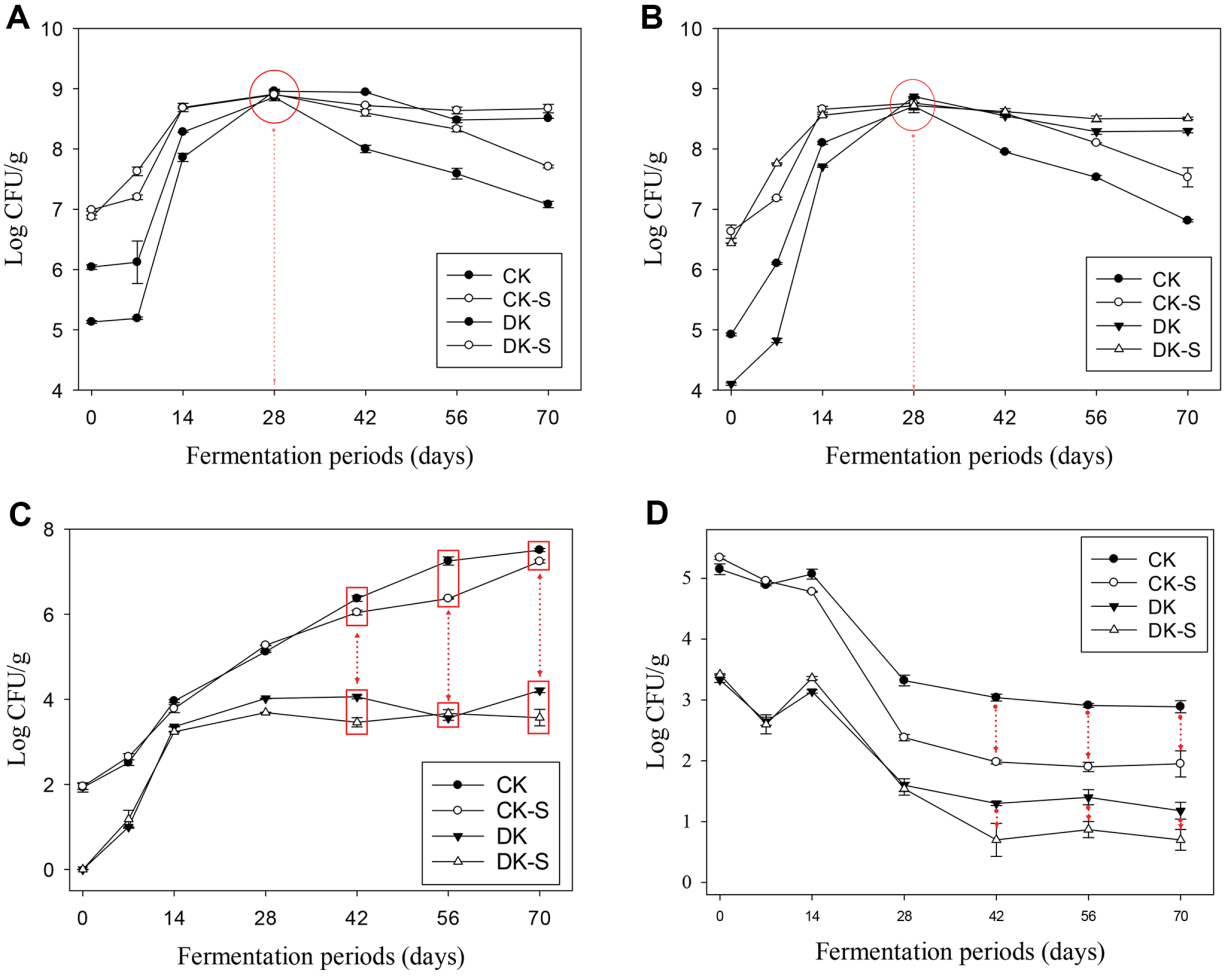
Changes in the counts of background microbiota in the four kimchi groups. (**A**) total aerobic bacteria, (**B**) lactic acid bacteria, (**C**) yeast and mold, and (**D**) total coliform counts. i) commercial kimchi (CK), ii) commercial kimchi supplemented with kimchi LAB starter without non-thermal treatment (CK-S), iii) microbial inactivated kimchi without kimchi LAB starter (DK), and iv) microbial inactivated kimchi supplemented with kimchi LAB starter (DK-S).

Changes in the microbiological populations of the four kimchi groups (CK, CK-S, DK, and DK-S) during fermentation were determined and are presented in Fig. 3. The initial TAB and LAB populations of the four groups under different treatment conditions were observed to differ by approximately 4.00 and 7.00 log (Fig. 3A and 3B). The numbers of TAB and LAB were higher in the CK-S and DK-S groups with *Leu. mesenteroides* WiKim32 than in the CK and DK groups without *Leu. mesenteroides* WiKim32 supplementation. The four groups of kimchi with different initial values of TAB and LAB showed a sharp increase in the numbers of both as the fermentation proceeded. After 28 days, the number of TAB in the four groups reached 8.91 ± 0.05 log and the number of LAB reached 8.73 ± 0.12 log. Although the initial populations were different, as fermentation progressed for 28 days, the populations of TAB and LAB increased simultaneously, showing a remarkable increase and no significant differences among the four groups. From the 28th day onwards, the number of TAB and LAB gradually decreased in the groups without microbial inactivated treatment, namely, CK and CK-S, while the microbial inactivated treatment groups, namely, DK and DK-S, maintained a level of 8.50 log up to 70 days without significant decreases. In the case of the number of yeasts/molds, approximately 2 log levels were detected in the CK and CK-S groups, but not in the microbial inactivated DK and DK-S groups (Fig. 3C). As the kimchi fermentation progressed, the number of yeasts/molds in the CK and CK-S groups gradually increased, and showed maximum levels of 7.51 ± 0.04 and 7.24 ± 0.04, respectively, in 70 days. In contrast, the number of yeasts/molds in the DK and DK-S groups, which were initially undetected, increased to 3.36 ± 0.07 and 3.24 ± 0.01, respectively, on the 14th day. However, between days 14 and 70, 4 log levels of yeasts/molds were maintained without any further increase. In the case of total coliforms, 5.24 ± 0.19 log levels were detected in the CK and CK-S groups, whereas 3.33 ± 0.11 log levels were detected in the DK and DK-S groups (Fig. 3D). The total coliform inactivation effect of thenon-thermal treatment was approximately 2 log. As fermentation progressed, the total coliform populations in the four groups showed a reduction of 2 log or more, indicating an additional inactivation effect on total coliforms by kimchi fermentation. Interestingly, the inactivation effect during kimchi fermentation was considerably greater in the CK-S and DK-S groups supplemented with *Leu. mesenteroides* WiKim32 compared to the CK and DK groups without *Leu. mesenteroides* WiKim32. Our results suggested that *Leu. mesenteroides* WiKim32 showed significant antimicrobial effects against total coliforms.

### Microbial Community Analysis

**Fig. 4:**
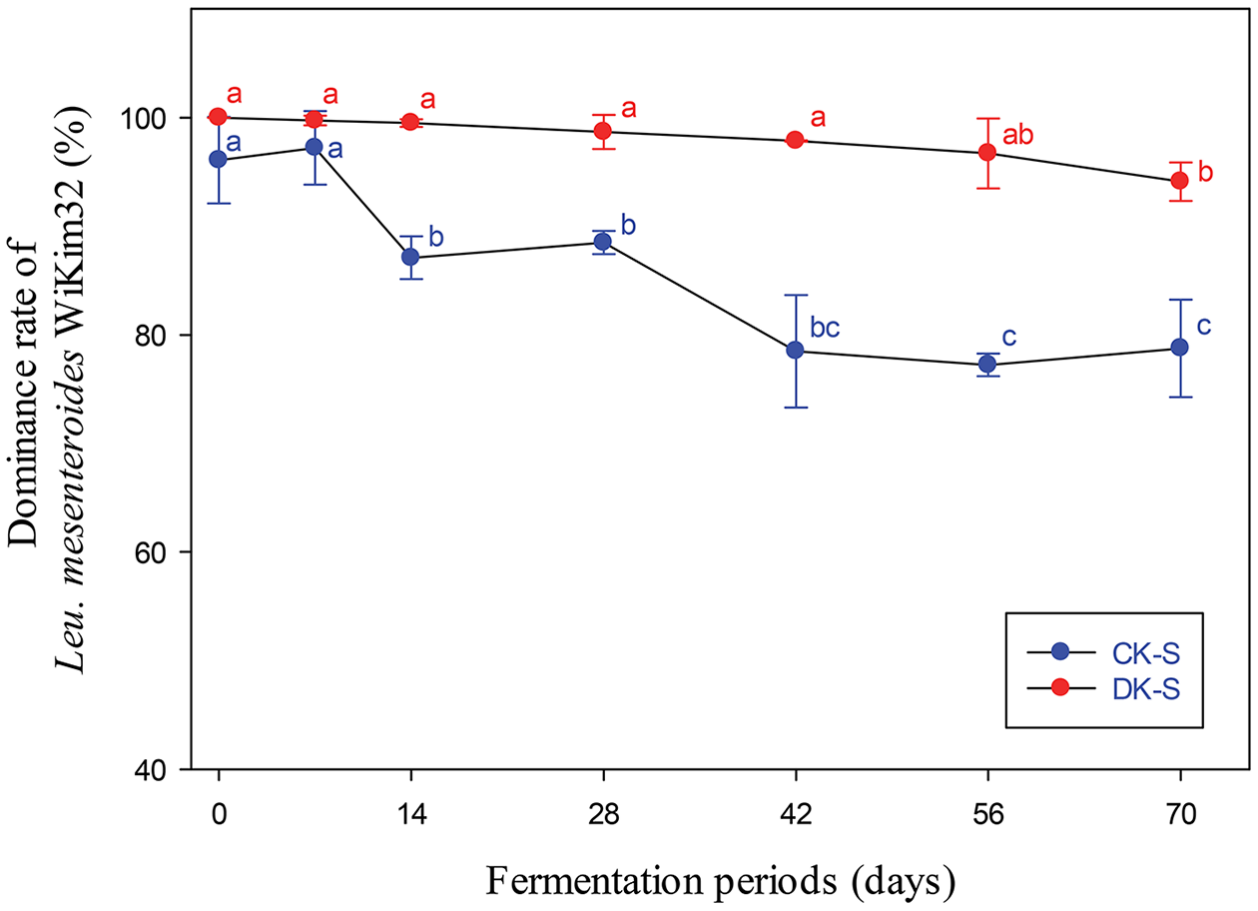
The ratio of *Leu. mesenteroides* WiKim32 cells to total viable bacteria in i) commercial kimchi supplemented with kimchi LAB starter without non-thermal treatment (CK-S) and ii) microbial inactivated kimchi supplemented with kimchi LAB starter (DK-S).

We analyzed whether *Leu. mesenteroides* WiKim32 was predominant during kimchi fermentation in the CK-S and DK-S groups supplemented with *Leu. mesenteroides* WiKim32 (Fig. 4). The CK-S group was commercial kimchi (CK) inoculated with *Leu. mesenteroides* WiKim32 and showed a significant decrease in *Leu. mesenteroides* WiKim32 dominance on the 14th day as fermentation progressed (87.1%) and then decreased gradually, showing a 76.19% dominance rate on the 70th day. The DK-S group inoculated with WiKim32 in sterilized kimchi (DK) showed *Leu. mesenteroides* WiKim32 as the dominant lactic acid bacterium in the kimchi microbial community at 94.1%, even after fermentation for 70 days. In particular, the 95% dominance rate was maintained without significant difference during 56 days of fermentation, which means that the initial microbial inactivation treatment can overcome the significant decrease in the starter dominance rate. Numerous previous studies have focused on analyzing the correlation between changes in microbial communities and changes in kimchi characteristics by adding kimchi LAB starters to general kimchi [8, 9, 25]. Although inoculation with certain starter strains can control kimchi fermentation, starter dominance can be affected by numerous external factors [6]. The main factors affecting the dominance rate of kimchi LAB starters include the amount of starter added, the microbiological environment of the initial kimchi, the superiority and inferiority of starters, fermentation conditions, and raw materials used. In this study, the microbiological environment of kimchi was optimized by obtaining kimchi through a pretreatment and sterilization process that targeted its raw materials. In addition, it was possible to considerably increase the dominance rate of the target LAB starters from the optimized environment (Fig. 4).

Taxonomic analysis based on bacterial 16S rRNA gene amplicon sequencing has demonstrated that fermented kimchi contains a distinct microbial community, with LAB, including *Leuconostoc*, *Lactobacillus*, and *Weissella* as the dominant species responsible for kimchi fermentation [24, 29-31]. Among the various dominant LABs, *Leuconostoc* spp. is known to be the dominant species during the early fermentation of kimchi [32]. In the present study, we evaluated the dominance rate of *Leu. mesenteroides* WiKim32 using a DNAzyme-based quantitative loop-mediated isothermal amplification (LAMP) assay [8, 9]. The DNAzyme-based quantitative LAMP assay developed in a previous study is a specialized method for analyzing the abundance ratio (the ratio of *Leu. mesenteroides* WiKim32 to total viable bacteria) of the kimchi starter. A previous study clearly distinguished *Leu. mesenteroides* WiKim32 from 72 other common LAB strains, including *Leuconostoc* strains, present in fermented kimchi [1, 33].

Commercially, *Leu. mesenteroides* have been used as kimchi LAB starters because of their enhanced effects on the sensory properties related to free sugar metabolism, particularly mannitol production [8, 9]. Although the composition of the LAB population or the dominance rate of the kimchi LAB starters depends on various external factors during the kimchi fermentation process; therefore, to control the kimchi fermentation environment for the clear dominance of the kimchi LAB starter, non-thermal sterilization treatment was applied during pre-processing of raw materials. Subsequent monitoring of the growth of *Leu. mesenteroides* WiKim32 in kimchi during fermentation confirmed the effect of non-thermal sterilization treatments in promoting *Leu. mesenteroides* WiKim32 dominance. The overwhelming dominance of *Leu. mesenteroides* WiKim32 in the DK-S group among the four groups is attributed to the effect of non-thermal sterilization.

**Fig. 5:**
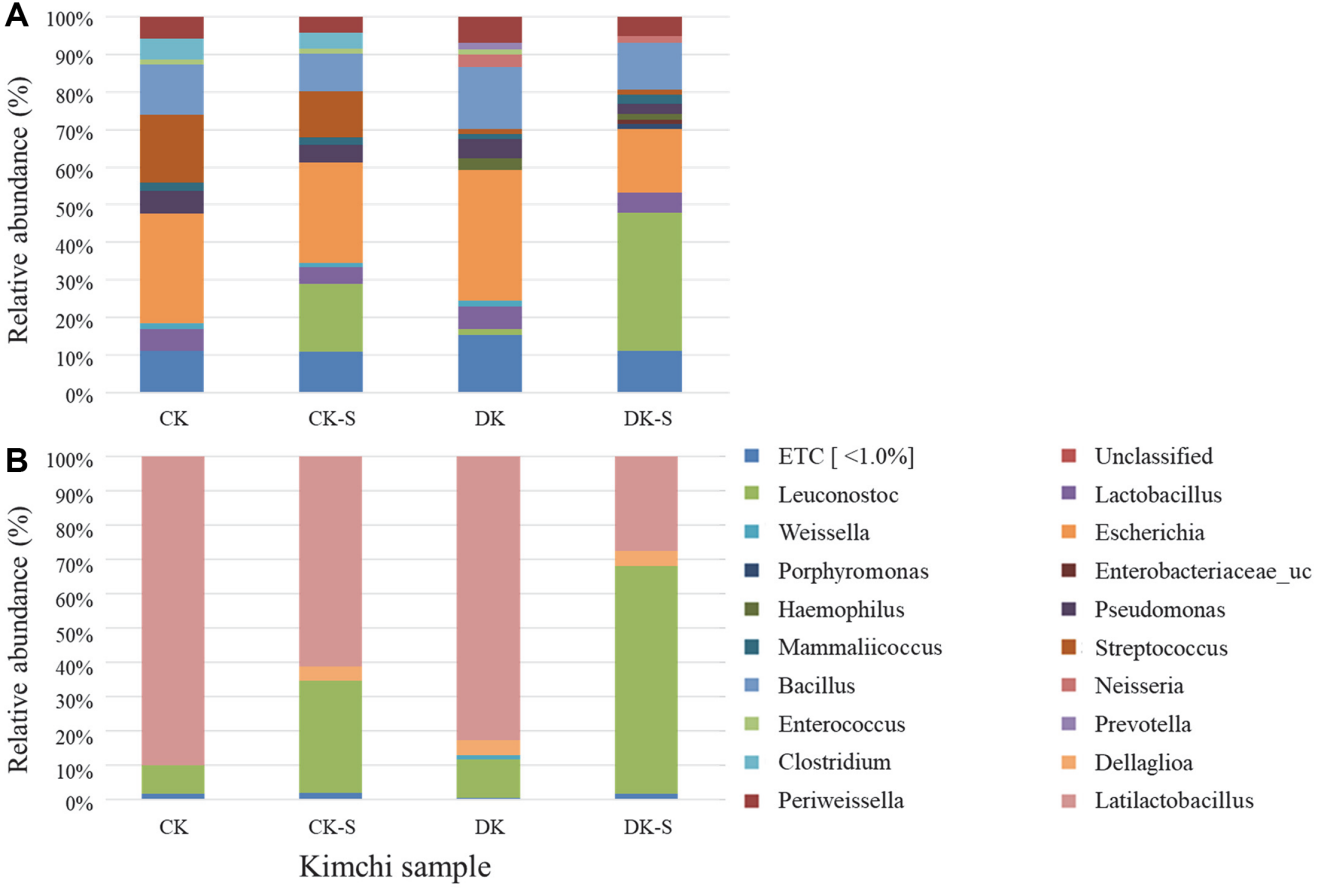
Relative abundance of total bacteria at the species level in the four kimchi groups: i) commercial kimchi (CK), ii) the commercial kimchi added with kimchi LAB starter without non-thermal treatment (CK-S), iii) microbial inactivated kimchi without kimchi LAB starter (DK), and iv) microbial inactivated kimchi supplemented with kimchi LAB starter (DK-S) at (**A**) day 0 and (**B**) day 70.

Changes in the microbial community of kimchi groups with different pretreatment conditions during fermentation at 4°C on days 1 and 70 are presented in Fig. 5A and 5B, respectively. The bacterial profiles of the kimchi groups were assessed using 16S rRNA sequencing at the species level. In the CK-S and DK-S groups supplemented with *Leu. mesenteroides* WiKim32 at the beginning of fermentation, the proportion of *the* *Leuconostoc genus* differed, accounting for approximately 17.87% and 36.7% of the total microbial communities, respectively, and similar microbial community patterns were observed in the other groups initially (Fig. 5A). The microbial composition over 70 days was similar in the CK and DK groups (Fig. 5B). However, there was a remarkable difference in the proportion of the *Leuconostoc genus* between the CK-S and DK-S groups supplemented with *Leu. mesenteroides* WiKim32 (Fig. 5B). This suggests the effectiveness of the sterilization pretreatment in securing the microbiological community ratio of the added LAB starters during kimchi fermentation. The reason for the difference in the proportion of *Leu. mesenteroides* (Figs. 4 and 5) are as follows. Sequence-based data (showing a substantial proportion of *Latilactobacillus*) represents the proportion of numerous microbial communities including lactic acid bacteria, and culture-based data (showing > 90% of isolates as *Leu. mesenteroides*) refers specifically to the distribution of colonies selected from the lactic acid bacteria medium. Therefore, differences arise in the relative proportions of the two. Numerous studies have reported that *Leu. mesenteroides* is the major LAB during the initial stage of kimchi fermentation at low temperatures, whereas *Lactobacillus sakei* (*L. sakei*) is mainly expressed during the middle and late fermentation stages [8, 9, 34, 35]. In the present study, in the CK, DK-S, and DK groups, changes in the proportion of *L. sakei* were similar to those observed in previous studies, with *L. sakei* accounting for a large proportion of the total microbial composition after 70 days. However, in the DK-S group, not only was the composition of *L. sakei* low but also the composition of *Leu. mesenteroides* was considerably relatively higher. Our results suggest that the use of *Leu. mesenteroides*, including inoculated *Leu. mesenteroides* WiKim32, as starter cultures after the *non-thermal* pretreatment could be considered more suitable than the starter culture inoculation process, which omits the commonly used non-thermal sterilization process.

### Changes in Free Sugar Content

Changes in free sugar content during fermentation of the four groups of kimchi samples were analyzed because the metabolite profiles of free sugars influencing kimchi flavor are closely related to the LAB clusters in kimchi. Initially (0 day), fructose, glucose, sucrose, and maltose were identified as the major free sugars contributing to the sweetness of kimchi; fructose was the most abundant, followed by glucose, sucrose, and maltose (Table 3), which is in line with the results of previous studies [2, 10]. Mannitol was not detected initially; however, it was identified as a major fermentation product as fermentation progressed. At the beginning of fermentation, glucose (in the range of 2193.55 and 2311.77 mg/kg), fructose (in the range between 3589.49 and 4030.90 mg/kg), sucrose (in the range between 261.51 and 346.24 mg/kg), and maltose (in the range between 110.87 and 159.39 mg/kg) but not mannitol, were detected (Table 3). After 70 days of fermentation, glucose, and fructose contents decreased over time to 82.79–901.81 mg/kg and 35.44–687.17 mg/kg, respectively, while mannitol content considerably increased (901.58–1517.88 mg/kg). Sucrose and maltose were not detected in any of the four kimchi groups after 70 days. *Leu. mesenteroides* WiKim32 likely expresses dextransucrase, which rapidly depletes sucrose concentrations, which has been shown to strongly affect sucrose levels [8, 9]. Mannitol was produced on day 14 (126.51–469.80 mg/kg) and can be produced by LAB utilizing sucrose, glucose, and fructose, is a naturally producing 6-carbon polyol and is usually found in various vegetables and fruits [36, 37]. Several polyols, such as mannitol, sorbitol, and xylitol, are potential alternative sweeteners for food [38]). In particular, *Leu. mesenteroides*, as a heterofermentative LAB, is known to generate high levels of mannitol by using sucrose, glucose, and fructose [39]. These data suggest that the addition of *Leu. mesenteroides* WiKim32 increased mannitol production, and enhanced the preferred umami flavor of kimchi.

### Identification of Volatile Compounds

**Fig. 6:**
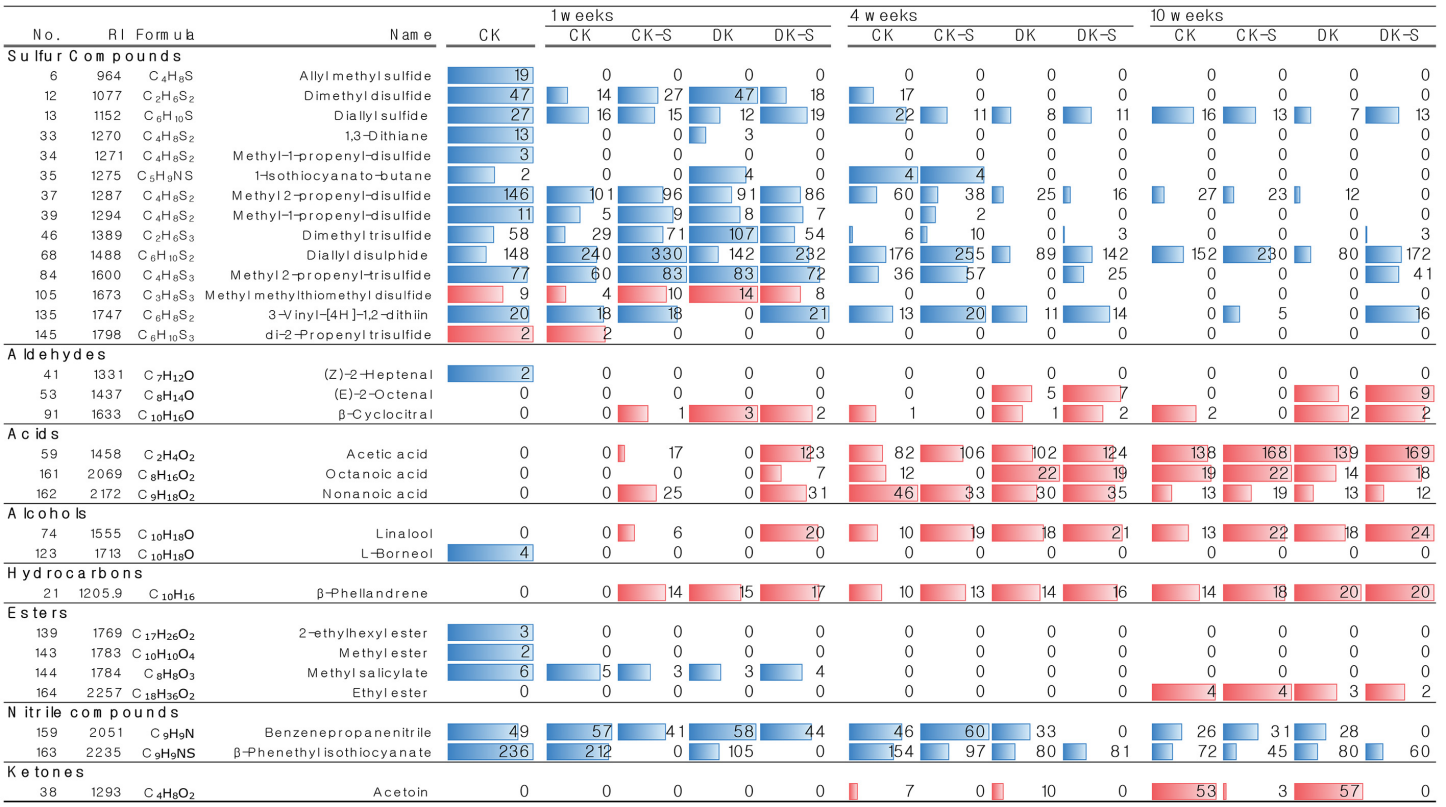
Volatile compounds identified from the four kimchi groups during kimchi fermentation. The red bar zone indicates an increasing pattern and the blue bar zone indicates a decreasing pattern with time. Commercial kimchi (CK), the commercial kimchi added with *Leu. mesenteroides* WiKim32 without non-thermal treatment (CK-S), microbial inactivated kimchi without *Leu. mesenteroides* WiKim32 (DK), and the microbial inactivated kimchi added with *Leu. mesenteroides* WiKim32 (DK-S).

The changes observed in VCs in the four groups of kimchi samples during the 70 days of kimchi fermentation are presented in Fig. 6. In total, 30 VCs were identified, 14 of which were sulfur compounds. The top five types of VCs according to the detection intensity of sulfur compounds were methyl 2-propenyl disulfide (garlic), diallyl disulfide (garlic, pungent, horseradish-like), methyl 2-propenyl-trisulfide(garlic), dimethyl trisulfide (raw cabbage), and dimethyl disulfide (sulfury, raw onion, and sour). The top five VCs accounted for the prominent volatile components, including fresh garlic, radish, and cabbage, that affect kimchi flavor. The other VCs were allyl methyl sulfide (sulfury, cooked meat), diallyl sulfide (pungent, garlic), methyl 1-propenyl-trisulfide (garlic), methyl 1-propenyl-disulfide (garlic), 1,3-dithiane, 1-isothiocyanato-butane, 3-vinyl [4H]-1,2-dithiin, di-2-propenyl trisulfide, and methyl methylthiomethyl disulfide [40]. The patterns of VC generation among the kimchi groups were analyzed using Microsoft Excel-based conditional formatting (Fig. 6). Most of the sulfur compounds showed the strongest intensity among the four groups immediately after manufacturing, and they decreased considerably during the fermentation process. A study on VC changes in sulfur compounds during kimchi fermentation showed a reduction in sulfur compounds caused by cysteine sulfoxide lyase, which increases as fermentation time increases [41]. The detection levels and changes in the pattern of sulfur compounds were similar to those reported in previous studies [42]. Furthermore, various VCs, including aldehydes, acids, alcohols, esters, nitrile compounds, hydrocarbons, and ketones, were also detected. Secondary metabolites such as aldehydes, alcohols, acids, and hydrocarbons, produced by heterofermentative bacteria, tend to increase as fermentation progresses. Several studies have reported that acetoin produced by genes encoding diacetyl reductase, acetolactate decarboxylase, and acetolactate synthase converting pyruvate to acetoin in *Leu. mesenteroides* subsp. mesenteroides is an important flavoring compound in kimchi [5, 42]. This indicates that *Leu. mesenteroides*are is responsible for the production of acetoin, which is related to the cheesy flavor intensity during kimchi fermentation [43, 44]. However, 2,3-butanediol dehydrogenase, which converts 2-acetoin to 2,3-butanediol with the regeneration of NAD^+^, was also found in the soft-core genome of *Leu. mesenteroides*are [41]. This finding suggests that this enzyme may attenuate cheese flavor intensity in kimchi fermented with *Leu. mesenteroides*are. Based on our results, the acetoin intensity of the kimchi groups supplemented with *Leu. mesenteroides* WiKim32 (CK-S and DK-S) was considerably lower than that of the groups to which *Leu. mesenteroides* WiKim32 were not added (CK and DK). Therefore, *Leu. mesenteroides* WiKim32 altered the content of the VC acetoin in kimchi groups.

## Conclusion

In this study, pretreatment based on sterilization processes, namely, SAEW treatment and UVC-LED irradiation of raw and subsidiary kimchi materials, reduced the initial microorganisms in raw and subsidiary kimchi materials, thereby increasing the efficiency and value of the kimchi LAB starter during fermentation. Pretreatment based on sterilization effectively reduced the number of initial microbial colonies in kimchi, thereby creating an environment in which kimchi LAB starters could better colonize and dominate, compared to non-sterilized kimchi. In addition, sterilization pretreatment effectively suppressed microorganisms that threaten the sanitary value and quality of kimchi. These differences in the initial microbial composition following sterilization pretreatment and the addition of kimchi LAB starters led to differences in the metabolites that affect the taste and flavor of kimchi produced in kimchi. The combined processing technology used in our study, namely, pre-sterilization and LAB addition, suggests a powerful approach for kimchi quality standardization. To improve the understanding of our findings, further studies should be conducted to investigate the effects of various kimchi LAB starters.

## Figures and Tables

**Fig. 1:**
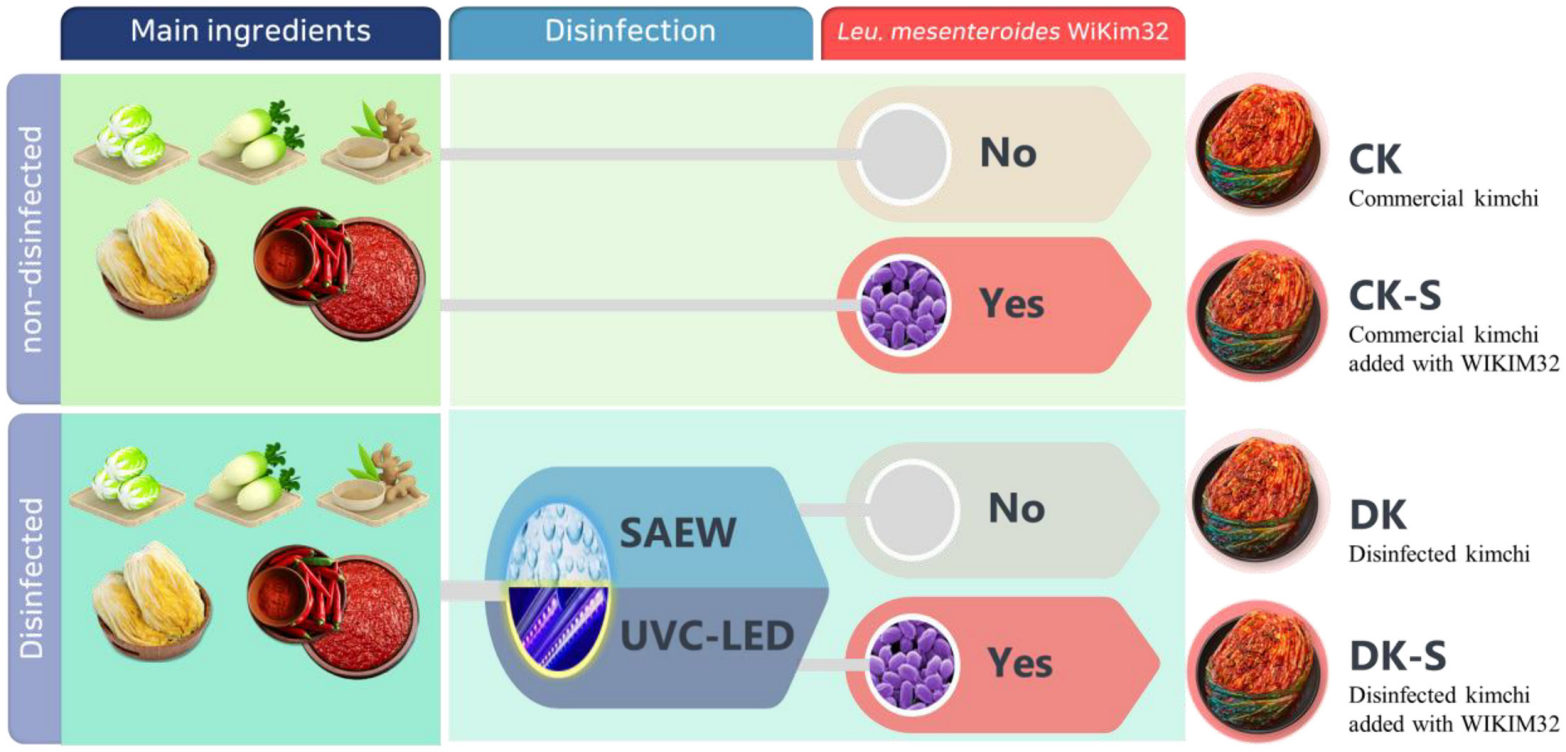
Illustration of each type of kimchi group.

**Table 1:** Reduction of total aerobic bacteria, lactic acid bacteria, yeast and mold, and total coliform counts before and after non-thermal treatment for microbial inactivation using slightly acidic electrolyzed water treatment of kimchi raw materials.

Treatment	Sample	Treatment	Total aerobic count	Lactic acid bacteria	Yeasts & mold	Total coliforms
SAEW^b^	Kimchi cabbage	Before disinfecting	5.52 ± 0.52	ND^a^	3.94 ± 0.48	2.95 ± 0.42
		After disinfecting	2.45 ± 0.17	ND	1.43 ± 1.03	ND
		Reduction value	3.07	-	2.51	2.96
	Brined cabbage	Before disinfecting	4.01 ± 0.34	3.80 ± 1.06	1.63 ± 0.21	2.93 ± 0.16
		After disinfecting	3.66 ± 0.30	1.54 ± 0.76	1.39 ± 0.12	1.66 ± 0.04
		Reduction value	0.34	2.26	0.25	1.27
	Radish	Before disinfecting	5.03 ± 0.23	2.35 ± 0.33	2.09 ± 0.30	1.84 ± 0.98
		After disinfecting	2.24 ± 0.35	1.44 ± 0.62	ND	ND
		Reduction value	2.79	0.91	0.79	1.84
	Garlic	Before disinfecting	6.65 ± 0.11	7.15 ± 0.19	ND	ND
		After disinfecting	6.03 ± 0.08	6.58 ± 0.29	ND	ND
		Reduction value	0.57	0.57	-	-
	Ginger	Before disinfecting	7.66 ± 0.17	4.42 ± 0.14	6.64 ± 0.25	4.29 ± 0.10
		After disinfecting	7.44 ± 0.19	3.47 ± 0.21	5.77 ± 0.23	3.34 ± 0.05
		Reduction value	0.22	0.95	0.87	0.95

^a^Not detectable. (the minimum detection level is 20 CFU/g), ^b^Slightly acidic electrolyzed water

**Table 2:** Reduction of total aerobic bacteria, lactic acid bacteria, yeast and mold, and total coliform counts before and after non-thermal treatment for microbial inactivation using UVC-LED irradiation of chili powder and Kimchi seasoning.

Treatment	Sample	Treatment	Total aerobic count	Lactic acid bacteria	Yeasts & mold	Total coliforms
UVC-LED^a^	Chili powder	Before disinfecting	5.85 ± 0.14	4.25 ± 0.33	4.01 ± 0.82	3.68 ± 0.30
		After disinfecting	5.48 ± 0.18	3.61 ± 0.05	3.18 ± 0.13	3.54 ± 0.13
		Reduction value	0.37	0.64	0.82	0.14
	Kimchi seasoning	Before disinfecting	6.99 ± 0.18	5.34 ± 0.16	2.94 ± 0.25	5.74 ± 0.20
		After disinfecting	5.19 ± 0.15	3.67 ± 0.18	1.47 ± 0.67	3.59 ± 0.03
		Reduction value	1.80	1.67	1.57	2.15

^a^Ultraviolet C light-emitting diode

**Table 3:** Changes in free sugar content of the four kimchi groups at 4°C for 70 days during fermentation.

Free sugars (mg/100 g)	Fermentation time (days)	Kimchi samples			
		CK	CK-S	DK	DK-S
Fructose	0	4,030 ± 23^Aba1)^	3,975 ± 34^Aa^	3,589 ± 52^Bb^	4,063 ± 49^ABa^
	7	4,017 ± 53^Aa^	4,107 ± 8^Aa^	3,694 ± 89^Ca^	3,830 ± 47^Bb^
	14	2,989 ± 26^Bb^	2,604 ± 88^Db^	3,539 ± 43^Ab^	2,830 ± 37^Cc^
	28	2,120 ± 141^Ac^	578 ± 41^Dc^	1,229 ± 22^Bc^	768 ± 28^Cd^
	56	339 ± 19^Cd^	332 ± 24^Ccd^	700 ± 20^Ad^	490 ± 21^Be^
	70	340 ± 13^Bd^	35 ± 3^Cd^	687 ± 16^Ad^	342 ± 10^Bf^
Glucose	0	2,193 ± 21^Ba^	2,311 ± 65^Aa^	2,193 ± 47^Ba^	2,208 ± 51^Ba^
	7	2,281 ± 8^Ab^	2,109 ± 18^Cb^	2,230 ± 20^Ba^	1,929 ± 23^Db^
	14	1,597 ± 14^Cc^	1,508 ± 4^Dc^	1,829 ± 8^Ab^	1,739 ± 12^Bc^
	28	1,162 ± 11^Cd^	1,095 ± 10^Dd^	1,445 ± 3^Ac^	1,298 ± 23^Bd^
	56	386 ± 10^De^	778 ± 33^Ce^	896 ± 34^Bd^	978 ± 22^Ae^
	70	319 ± 11^Cf^	82 ± 5^Df^	718 ± 24^Be^	901 ± 2^Af^
Sucrose	0	324 ± 20^Aa^	346 ± 16^Aa^	266 ± 4^Bb^	231 ± 16^Ca^
	7	239 ± 27^Bb^	282 ± 7^Ab^	302 ± 4^Aa^	144 ± 6^Cb^
	14	126 ± 4^Bc^	36 ± 1^Dc^	139 ± 12^Ac^	50 ± 6^Cc^
	28	83 ± 9^Ad^	^a^ND	84 ± 5^Ad^	ND
	56	ND	ND	ND	ND
	70	ND	ND	ND	ND
Maltose	0	123 ± 10^Ba^	159 ± 2^Aa^	110 ± 7^Ba^	119 ± 19^Ba^
	7	86 ± 12^Bb^	110 ± 4^Ab^	93 ± 8^Bb^	121 ± 13^Aa^
	14	39 ± 3^Bc^	34 ± 1^Cc^	64 ± 2^Ac^	30 ± 1^Db^
	28	ND	ND	ND	ND
	56	ND	ND	ND	ND
	70	ND	ND	ND	ND
Mannitol	0	ND	ND	ND	ND
	7	ND	ND	ND	ND
	14	174 ± 4^Cd^	379 ± 9^Bd^	126 ± 1^Dd^	469 ± 5^Ad^
	28	536 ± 9^Cc^	1,073 ± 12^Ac^	486 ± 5^Dc^	1,048 ± 8^Bc^
	56	715 ± 0^Db^	1,378 ± 14^Ab^	830 ± 18^Cb^	1,224 ± 33^Bb^
	70	935 ± 27^Ca^	1,517 ± 14^Aa^	901 ± 35^Ca^	1,310 ± 11^Ba^

^a^ND: Not detectable.

All values represent the mean ± SD. Means sharing different letters in the same column (a–f) and row (A-D) are significantly different (*p* < 0.05).

Commercial kimchi, CK; the commercial kimchi added with *Leu. mesenteroides* WiKim32 without non-thermal treatment, CKS; microbial inactivatedkimchi without *Leu. mesenteroides* WiKim32, DK; microbial inactivated kimchi supplemented with *Leu. mesenteroides* WiKim32, DK-S.
